# *Eryngium foetidum* L. Essential Oils: Chemical Composition and Antioxidant Capacity

**DOI:** 10.3390/medicines4020024

**Published:** 2017-04-28

**Authors:** Paul S. Thomas, Emmanuel E. Essien, Samuel J. Ntuk, Mohammad I. Choudhary

**Affiliations:** 1 Department of Pharmacognosy and Natural Medicine, University of Uyo, Uyo 520101, Nigeria; 2 Department of Chemistry, University of Uyo, Uyo 520101, Nigeria; emmanuelessien@uniuyo.edu.ng (E.E.E.); samkelly202@yahoo.com (S.J.N.); 3 Hussain Ebrahim Jamal Research Institute of Chemistry, International Centre for Chemical and Biological Sciences, University of Karachi, Karachi 75270, Pakistan; hej@cyber.net.pk

**Keywords:** Apiaceae, *Ergnium foetidum*, essential oils, antioxidant activity

## Abstract

**Background:** *Eryngium foetidum* essential oils from Nigeria were investigated for the first time in order to ascertain their potency as natural antioxidants. *E. foetidum* is an aromatic and medicinal herb used in ethno-medicine and as a traditional spice for foods. **Methods:** The hydro-distilled oils of *E. foetidum* were subjected to gas chromatography-mass spectrometry (GC-MS) analysis. Free radical scavenging capacity of the volatile oils was determined using 2, 2-diphenyl-1-picrylhydrazyl (DPPH) and ferric reducing antioxidant power (FRAP) assays. **Results:** Leaf volatile oil contained a high proportion of (*E*)-2-Dodecenal (28.43%), 13-tetradecenal (27.45%), dodecanal (14.59%) and 2,4,5-trimethylbenzaldehyde (10.77%); the stem oil comprised of dodecanal (20.21%), 2,4,5-trimethylbenzaldehyde (18.43%) and (*E*)-2-dodecenal (8.27%), while 2,4,5-trimethylbenzaldehyde (56.08%), 13-tetradecenal (9.26%) and (*E*)-2-dodecenal (7.65%) were the most dominant in the root oil. The IC_50_ values for the leaf, stem and root oils were 56 µg/mL, 46µg/mL and 54.5 µg/mL respectively in the DPPH assay while the leaf oil exhibited the highest reducing potential among the test oils in the FRAP assay. **Conclusions:** The Nigerian *E. foetidum* volatile oils contain high amount of acyclic aldehydes and aromatic compounds. The oils are a potential source of natural antioxidant as demonstrated by their strong antioxidant activity.

## 1. Introduction

*Eryngium* L. (Apiaceae) is comprised of approximately 250 species reputed as potential pharmaceutical crops based on their phytochemical constituents and pharmacological potential [1]. *E. foetidum* is an herb indigenous to Central America and West Indies [2]. The leaves are often substituted for coriander leaves due to its similar pungent smell [3]. *E. foetidum* is employed in the treatment of diabetes, rheumatism, several anti-inflammatory, respiratory (cold, asthma, cough, sinusitis), and stomach disorders [4,5,6].

Free radicals are involved in the etiology of several degenerative ailments in humans. The unique biological activities of essential oils have been attributed to the mosaic combination of constituents. Studies on *E. foetidum* essential oils from diverse geographic climes of the world seem to indicate chemical variability in the proportion and occurrence of aromatic and acyclic aldehydes. A number of researchers have reported (*E*)-2-dodecen-1-al as the main constituent of *E. foetidum* leaf oil from Malaysia, Bangladesh and Vietnam, but low amount in the Cuban oil [2,7,8,9,10]. Martins et al. [5] showed the leaf oil of *E. foetidum* comprise of 2,3,6-trimethylbezaldehyde (5.5%–23.7%), (*E*)-2-dodecenal (15.9%–37.5%) and (*E*)-2-tetradecenal (18.7%–25.3%) while the Columbian sample predominated in (*E*)-2-dodecenal, 5-dodecene, tetradecanal, tetradecenal, 2,3,5-trimethylbenzaldehyde and trimethylphenol [6]. In addition, a number of *Eryngium* species have been investigated for their essential oil composition, such as *E. tricuspidatum*, *E. bungei*, *E. duriaei*, *E. billardieri*, *E. caeruleum* and *E. corniculatum* [11,12,13,14,15,16].

The anti-oxidant and anti-proliferative activities of *E. foetidum* leaf volatile oils from Columbia and India have been reported [6,17]. Similarly, *E. tricuspidatum* essential oil was shown to exhibit both antimicrobial and antioxidant activities [11]. In this communication, we present the first report on the composition and antioxidant activity of the leaf, stem and root essential oils of the Nigerian grown *E. foetidum*.

## 2. Materials and Methods

### 2.1. Plant Sample

The leaf, stem and root of *E. foetidum* were collected from mature plants cultivated in Uyo Local Government Area of Akwa Ibom State, Nigeria, in the month of May 2016. The Sample was identified by a taxonomist in the Department of Botany and Ecological Studies, University of Uyo, where voucher specimen was deposited. The essential oils were obtained by hydrodistillation (4 h) of the fresh plant parts using a Clevenger-type apparatus in accordance with the British Pharmacopoeia [18]. The oils were dried over sodium sulfate and stored in refrigeration (4 °C) after estimation of percentage yield.

### 2.2. Gas Chromatography—Mass Spectrometry (GC–MS)

The volatlie oils were subjected to GC-MS analysis on an Agilent system consisting of a model 7890 N gas chromatograph, a model mass detector Triple Quad 7000 A in EI mode at 70 eV (*m*/*z*) range 40–600 amu) (Agilent Technologies, Santa Clara, CA, USA), and an Agilent ChemStation data system. The GC column was an HP-5 ms fused silica capillary with a (5% phenyl)-methyl polysiloxane stationary phase (30 m × 250 μm × 0.25 μm). The carrier gas was helium with a column head pressure of 9.7853 psi and flow rate of 1.2 mL/min. Inlet temperature and MSD detector temperature was 250 °C. The GC oven temperature program was used as follows: 50 °C initial temperature, held for 5 min; increased at 6 °C/min to 190 °C for 20 min; increased 7 °C/min to 290 °C for 15 min; increased 7 °C/min to 300 °C for 10 mins. The sample was dissolved in dichloromethane, and 2 µL was injected (split ratio 10:1; split flow 12 mL/min).

The components were identified by comparison of their mass spectra with NIST 1998 library data of the GC-MS system as well as by comparison of their retention indices (RI) with the relevant literature data [19]. The relative amount of each individual component of the essential oil was expressed as the percentage of the peak area relative to the total peak area. RI value of each component was determined relative to the retention times of a homologous n-alkane series with linear interpolation on the HP-5 ms column.

### 2.3. Antioxidant Activity

#### 2.3.1. DPPH Radical Scavenging Activity

The DPPH free radical scavenging of the *E. foetidum* essential oils and ascorbic acid prepared in methanol at concentrations (20–100 µg/mL) were evaluated according to the method of Shekhar and Anju [20]. 1 mL of 0.1 mM DPPH solution in methanol was added to 3 mL the solutions prepared with the oils and standard, and stirred for 1 min. Each mixture was kept in the dark at room temperature for 30 min and the absorbance recorded against a blank at 517 nm. The assays were carried out in triplicate and the results expressed as mean values ± standard deviation. Lower absorbance of the reaction mixture indicated higher free radical activity. Percentage scavenging activity was calculated using the expression:

$$\%\;\mathrm{Scavenging}\;\mathrm{activity}=\frac{Absorbance\;of\;Control-Absorbance\;of\;Sample}{Absorbance\;of\;Control}\times 100$$

#### 2.3.2. FRAP Assay

The reducing power of the essential oils was determined according to the method of Oyaizu [21]. Various concentrations (20, 40, 60, 80 and 100 µg/mL) of essential oils and ascorbic acid were mixed with phosphate buffer (2.5 mL, 0.2 M, pH 6.6) and 1% (*w*/*v*) of potassium ferricyanide water solution (2.5 mL). The mixture was incubated at 50 °C for 20 min. Aliquots of trichloroacetic acid (2.5 mL, 10%, aqueous solution (*w*/*v*)) were added to the mixture and centrifuged at 3000 rpm for 10 min. The supernatant (2.5 mL) was mixed with distilled water (2.5 mL) and a freshly prepared ferric chloride solution (0.5 mL, 0.1% (*w*/*v*)). After 30 min of incubation at room temperature in the dark, the absorbance of the solution was measured at 700 nm. The experiment was performed in triplicate and the average absorbance noted for each measurement. Higher absorbance indicates higher reducing power. The ferric-reducing capacity of the essential oils and standard compound were expressed graphically by plotting the absorbance against concentration.

## 3. Results and Discussion

The yields of the leaf, stem and root essential oils of *E. foetidum* were 0.2%, 0.16% and 0.17% respectively. The characterization of *E. foetidum* oils are presented in Table 1. Thirty four (34) constituents were identified in the volatile oils accounting for 99.99%, 96.63% and 92.27% respectively. The analyzed oils were qualitatively and quantitatively different, characterized by high amount of aromatic, linear unsaturated and saturated aldehydes. The leaf volatile oil contained a high proportion of (*E*)-2-Dodecenal (28.43%), 13-tetradecenal (27.45%), dodecanal (14.59%) and 2,4,5-trimethylbenzaldehyde (10.77%); the stem oil comprised of dodecanal (20.21%), 2,4,5-trimethylbenzaldehyde (18.43%) and (*E*)-2-dodecenal (8.27%), while 2,4,5-trimethylbenzaldehyde (56.08%), 13-tetradecenal (9.26%) and (*E*)-2-dodecenal (7.65%) were the most dominant in the root oil. The major aldehyde compounds occurred in all the samples, except 13-tetradecenal detected in both leaf and root oils. Monoterpene hydrocarbons occurred in the range 5.49%–28.11%, while the oxygenated monoterpene (3.49%) and sesquiterpene hydrocarbon (0.44%) were detected in small quantities in the leaf part only. 

The comparison of our findings with volatile oils of *E. foetidum* from other regions reveal some similarities and differences which may attributed to a number of factors such as clime, plant maturity and variety, processing and methods of analysis. (*E*)-2-Dodecenal, the major component (28.43%) of the leaf oil in our Nigerian sample was reported predominant in volatile oils from Vietnam (45.5%) [8], Malaysia (59.7%) [2], S. Tome (15.7 & 37.5%) [5], and Bangladesh (37.4%) [10]; but detected in small amount in the leaf oils of Cuba (5.7%) [9] and Taiwan [22]. Similarly, 2,4.5-trimethylbenzaldehyde (lauraldehyde) (10.77%) in the leaf oil (Table 1) was presented as a major component (20.5%) in the Cuban oil [9], Venezuelan Andes (27.7%) [23] and Bangladesh (5.1%) [10]. On the other hand, its positional isomer, the 2,3,6-trimethylbenzaldehyde was not detected in the Nigerian oils, but was present as a dominant component (23.7%) in the S. Tome [5] and Malaysian (9.6%) [2] leaf sample. The 13-tetradecenal (27.45%) in the leaf oil (Table 1) was not identified in S. Tome leaf oil, however the isomer, (*E*)-2-tetradecenal (18.7 & 25.3%) was reported as a major constituent [5]. The 2,3,6-trimethylbenzaldehyde (37.55%) and 2-formyl-1,1,5-trimethylcyclohexa-2,4-diene-6-ol (19.82%) reported in the Malaysian root essential oil [2] was not identified in Nigerian root oil. Literature has revealed that the stem essential oil of *E. foetidum* is reported for the first time, however the Cuban seed oil is reported to contain carotol (19.3%) and hexadecanoic acid (12.0%) among other constituents [9]. Eyres et al. [24] showed that the prominent “character-impact” factor of *E. foetidum* essential oil are (*E*)-2-dodecenal and (Z)-2-dodecenal.

**Figure 1:**
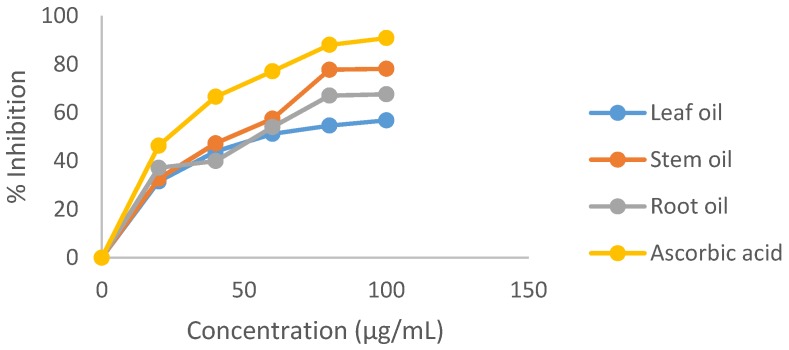
DPPH radical scavenging activity of *E. foetidum* essential oils.

The DPPH radical scavenging activity of *E. foetidum* essential oils is depicted in Figure 1. The plot indicates the scavenging ability of the oils as percent inhibition at various concentrations; the scavenging effect was concentration dependent. This was demonstrated by the oils ability to act as hydrogen atoms or electrons donor in the conversion of the stable purple coloured DPPH to the reduced yellow coloured DPPH-H. Ascorbic acid (100 µg/mL) showed the highest percent inhibition (90.79%), followed by the stem oil (78.08%), root oil (67.53%) and leaf oil (56.76%). DPPH radical activity is usually presented with their IC_50_ value. The IC_50_ values for stem, root and leaf oils were 46 µg/mL, 54.5 µg/mL and 56 µg/mL respectively while ascorbic acid showed 22 µg/mL.

The chemical composition of the stem oil (which exhibited the highest DPPH radical scavenging effect) reveals a number of oxygenated constituents that were not identified in both leaf and root volatile oils, such as 2,4,6-trimethyl phenol, 2-undecanol, 1-undecanol, 1-dodecanol, (*E*)-2-dodecen-1-ol and other aliphatic aldehyde compounds. These compounds may have furnished the antiradical activity shown by the stem oil. Therefore, the antioxidant activity depends on chemical composition. Chandrika et al. [17] reported a higher DPPH radical activity (96.674% inhibition at 50 µg/mL, IC_50_ value of 22.14) for the Indian *E. foetidum* leaf oil compared with the Nigerian leaf oil (Table 1); however, the authors did not present the chemical profile of the leaf oil. Previous investigation on *E. foetidum* leaf oils from India revealed the occurrence of muurola-4,10(14)-diene-1-ol (10.2%), hexahydrofarnesylacetone (5.5%), palmitic acid (4.6%) and phytol (4.9%) which were not identified in the Nigerian samples [25].

**Figure 2:**
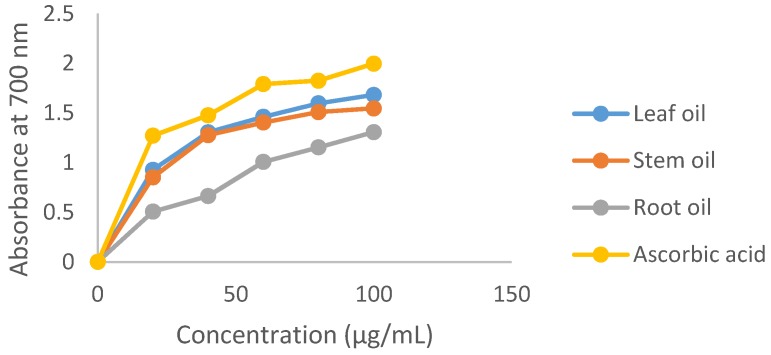
Ferric ion reducing capacity of *E. foetidum* volatile oils.

The dose dependent ferric reducing power of *E. foetidum* essential oils and ascorbic acid is presented in Figure 2. The FRAP assay is an indication of the reducing potential of the antioxidants against the oxidative consequences of reactive oxygen species. It was observed that the reducing capacity of the volatile oils and standard compound increased with a corresponding increase in concentration. The leaf, stem and root oils demonstrated good reducing effects (20–100 µg/mL: 0.927–1.682, 0.852–1.546 and 0.506–1.307 respectively). This implicates the oil constituents’ ability to reduce the (Fe^3+^) to (Fe^2+^) by electron transfer. The ferric ion reduction ability of ascorbic acid in the assay (20–100 µg/mL, 1.271–1.996) was relatively higher than the absorbance values for the volatile oils. The trend of reducing power (100 µg/mL) followed the order: ascorbic acid > leaf oil > stem oil > root oil. Merghache et al. [11] also showed that *E. tricuspidatum* essential oil exhibited a dose dependent ability (to reduce Fe^3+^ to Fe^2+^) in the FRAP assay.

## 4. Conclusions

The analyzed volatile oils from the leaf, stem and root parts of *E. foetidum* mainly consist of aliphatic and aromatic compounds. The strong antioxidant activity exhibited by the *E. foetidum* volatile oils serves as a substantial basis for their use in ethno-medicine and as potential antioxidant for preventing oxidative deterioration in foods.

## Figures and Tables

**Table 1:** Composition of *E. foetidum* essential oils.

**Compound**	**KI**	**Leaf (%)**	**Stem (%)**	**Root (%)**	**QI (%)**
n-Octane	800	-	0.24	-	96
n-Nonane	900	-	0.44	-	98
α-Pinene	938	0.44	8.27	2.64	95
β-Pinene	983	-	3.59	-	98
Sabinene	971	-	0.46	-	99
n-Decane	1000	-	0.38	-	97
α-Phellandrene	1006	-	0.30	-	90
Benzene-1,2,3-trimethyl	1020	0.79	0.92	1.67	90
p-Cymene	1024	1.22	6.46	6.94	99
Limonene	1028	-	7.98	-	95
γ-Terpinene	1058	3.83	1.05	4.05	99
Undecane	1100	0.3	1.66	-	99
Nonanal	1104	-	0.57	-	98
Decanal	1204	2.57	6.41	-	98
2,4,6-Trimethylphenol	1241	-	0.96	-	96
1-Decanol	1258	0.5	0.5	-	97
2-Undecanol	1287	-	0.27	-	98
Thymol	1292	3.49	-	-	98
Tridecane	1300	-	0.66	-	98
Undecanal	1303	-	1.73	-	97
2,4,5-Trimethylbenzaldehyde	1342	10.77	18.43	56.08	97
1-Undecanol	1357	-	0.55	-	96
**Compound**	**KI**	**Leaf (%)**	**Stem (%)**	**Root (%)**	**QI (%)**
Dodecanal	1412	14.59	20.21	1.0	97
β-Caryophyllene	1414	0.44	-	-	99
β-Cedrene	1442	-	-	0.76	95
1-Dodecanol	1457	-	2.58	-	96
2-Dodecenal	1460	28.43	8.27	7.65	97
(*E*)-2-Dodecen-1-ol	1465	-	1.47	-	95
2,4,6-Trimethylbenzoic acid	1490	-	-	0.69	95
Tridecanal	1505	-	0.56	-	94
(*E*)-2-Tridecenal	1510	-	1.71	-	94
Diepicedrene-1-oxide	1548	1.11	-	0.82	90
13-Tetradecenal	1608	27.45	-	9.26	93
Tetradecanal	1611	4.06	-	0.71	97
Monoterpene hydrocarbons		5.40	28.11	14.39	
Oxygenated monoterpenes		3.49	-	-	
Sesquiterpene hydrocarbons		0.44	-	-	
Oxygenated sesquiterpenes		-	-	-	
Other constituents		90.57	68.52	77.88	
Total identified		99.99	96.63	92.27	

KI = Kovat’s index in order of elution on HP-5ms column; QI, “quality index”, indicates the fit comparison of experimental mass spectrum and NIST library spectrum; - = Not detected.
